# Advancing Traditional Dunhuang Regional Pattern Design with Diffusion Adapter Networks and Cross-Entropy

**DOI:** 10.3390/e27050546

**Published:** 2025-05-21

**Authors:** Yihuan Tian, Tao Yu, Zuling Cheng, Sunjung Lee

**Affiliations:** 1 Department of Space and Culture Design, Kookmin University, Seoul 02707, Republic of Korea; tianyihuan@kookmin.ac.kr; 2 Department of Smart Experience Design, Kookmin University, Seoul 02707, Republic of Korea; ytkmu0517@kookmin.ac.kr; 3 Department of Global Convergence, Kangwon National University, Chuncheon-si 24341, Republic of Korea; 9710136280046@kangwon.ac.kr

**Keywords:** Dunhuang patterns, pattern generation, cross-entropy loss, diffusion model, intelligent generation, attention mechanism

## Abstract

To promote the inheritance of traditional culture, a variety of emerging methods rooted in machine learning and deep learning have been introduced. Dunhuang patterns, an important part of traditional Chinese culture, are difficult to collect in large numbers due to their limited availability. However, existing text-to-image methods are computationally intensive and struggle to capture fine details and complex semantic relationships in text and images. To address these challenges, this paper proposes the Diffusion Adapter Network (DANet). It employs a lightweight adapter module to extract visual structural information, enabling the diffusion model to generate Dunhuang patterns with high accuracy, while eliminating the need for expensive fine-tuning of the original model. The attention adapter incorporates a multihead attention module (MHAM) to enhance image modality cues, allowing the model to focus more effectively on key information. A multiscale attention module (MSAM) is employed to capture features at different scales, thereby providing more precise generative guidance. In addition, an adaptive control mechanism (ACM) dynamically adjusts the guidance coefficients across feature layers to further enhance generation quality. In addition, incorporating a cross-entropy loss function enhances the model’s capability in semantic understanding and the classification of Dunhuang patterns. The DANet achieves state-of-the-art (SOTA) performance on the proposed Diversified Dunhuang Patterns Dataset (DDHP). Specifically, it attains a perceptual similarity score (LPIPS) of 0.498, a graph matching score (CLIP score) of 0.533, and a feature similarity score (CLIP-I) of 0.772.

## 1. Introduction

Image generation based on deep learning has been a widely researched topic in the field of computer vision. It focuses on generating various types of images based on the provided modal conditions. For example, text-to-image generation create semantically consistent patterns based on natural language descriptions. Applying deep learning to the generation of Dunhuang patterns not only supports the preservation and inheritance of traditional culture, but also offers rich inspiration and resources for modern design. From a design studies perspective, this technology enables designers to conduct an unprecedented in-depth exploration of traditional visual languages, thereby deconstructing, recombining, and innovating these historical motifs within contemporary design contexts. Furthermore, the application of artificial intelligence in this domain can foster a new synergy between algorithmic generative capabilities and the designer’s creative intuition, leading to innovative design outcomes that are both culturally resonant and forward-looking. Dunhuang patterns have great application prospects in the fields of virtual reality, computer-aided design, unified generation patterns, and style migration. Deep learning can analyze the data and feature extraction of Dunhuang patterns to generate corresponding datasets. This not only improves the accuracy of pattern recognition but also speeds up the construction of digital pattern libraries. In addition, cross-entropy loss is a key component in deep learning network training. It helps the model learn and optimize by minimizing the gap between the predicted probability distribution and the true distribution, thereby improving performance and generalization. In summary, AI can generate patterns of the same style based on the dataset, providing new possibilities in the field of creative design.

Image generation not only creates new visual content from existing images but also generates entirely new images based on different types of data, such as text, sketches, and scene graphs [1]. The development of this technique is important for several application areas, such as data enhancement and pattern recognition [2]. To address the problem that a single input image may correspond to multiple outputs, Jun-Yan Zhu et al. [3] modeled the distribution of possible outputs using a conditional generative model. They mapped the ambiguities into low-dimensional latent vectors and randomly sampled them during testing. This method effectively prevents the pattern collapse problem. It improves the diversity of results by explicitly encouraging a bidirectional mapping between outputs and potential latent codes. To generate photo-realistic images from semantic descriptions, a hierarchically nested adversarial objective was introduced. This objective helps standardize intermediate representations and assists the generator in capturing complex image statistics. This approach improves semantic consistency and image fidelity by using a multi-stage generator architecture. It also employs a multi-purpose adversarial loss, which increases the efficient use of both image and text information [4]. SPADE [5] is used to generate realistic images from semantic layouts. Traditional network architectures often struggle to process semantic masks effectively. To address this, a spatially adaptive normalization layer is proposed. It modulates the activations in the normalization layer through spatially adaptive transformations, allowing semantic information to be conveyed more efficiently. This method demonstrates its superiority on several challenging datasets and supports multimodal and style-guided image synthesis. A new evaluation metric, Fréchet Joint Distance (FJD) [6], has been proposed for evaluating conditioned generative adversarial networks (CGANs). FJD implicitly measures image quality and condition consistency. It does so by calculating the Fréchet distance between the joint distribution of the image and its condition, as well as internal condition diversity and other properties. This metric provides a promising unified metric for CGAN model selection and benchmarking. These research efforts [7,8,9] have promoted the development of image generation techniques. They also offer new perspectives and tools for evaluating and comparing different models. In China, a deep learning-based image restoration technique applies generative adversarial networks (GANs) to restore damaged textile images excavated from Chu tombs [10]. This approach avoids direct contact with the artifacts, reducing the risk of damage, while also providing a new avenue for artifact restoration and research. There are several challenges when applying image generation to Dunhuang patterns. The main ones include accurately recovering complex and irregular textures, ensuring the coherence and consistency of the reconstructed pattern, and accurately predicting missing details when substantial portions of the original are unavailable.

To address the above challenges, this paper proposes the Diffusion Adapter Network. The model provides structural guidance to a large-scale text-to-image model, enabling multimodal Chinese traditional pattern generation. In the field of design science, this study combines deep learning with diffusion model innovation. It opens up a new path for the preservation, innovation, and modern design application of traditional cultural symbols. At the theoretical level, this study introduces advanced AI technologies to enhance design science, challenge traditional design frameworks, and explore new creative directions. Taking Dunhuang pattern generation as an example, we propose the Diffusion Adapter Network (DANet), which effectively integrates multimodal information. This model enables the efficient generation of complex traditional patterns and provides new theoretical perspectives and methodologies for design studies. In practice, this method offers designers a powerful tool to deeply explore the meaning of traditional cultural symbols, accurately extract key features, and reinterpret them in a modern context. It not only helps to supports the preservation of endangered patterns, but also stimulates creative inspiration, fosters culturally rich and innovative designs, and promotes diversity in the design industry. This paper has four main contributions:

(1) We propose a new model, DANet, which integrates textual input with sketch information to efficiently generate Dunhuang patterns. The multihead attention mechanism in the attention adapter processes multiple subspaces in parallel, with each subspace focusing on different features. This allows the model to capture both global structures and fine-grained details simultaneously, enhancing the accuracy and precision of the generated patterns.
(2) In order to extract multiscale feature information of a Dunhuang image, the MSAM module simultaneously considers features at different resolutions to provide a more comprehensive image understanding. It not only recognizes large contours and shapes but also captures small details and textures.
(3) The ACM module adaptively adjusts the generation guidance coefficients of different feature layers. This dynamic balancing improves the contribution of different feature layers, improving both the accuracy and efficiency of the generation process.
(4) For the Dunhuang pattern generation task, cross-entropy loss is introduced as an auxiliary supervision signal to enhance the semantic understanding capability of the attention adapter. This compensates for the limited semantic learning caused by freezing the parameters of the diffusion model. As a result, the generated Dunhuang images exhibit improved semantic consistency and accuracy.

## 2. Related Work

High-quality generative adversarial network (GAN)-based picture generating models are popular. The primary research studies, enhancement mechanisms, and characteristics of GAN-based high-quality image creation techniques are first compiled and concluded. Following that, we provide a new diffusion model for producing high-quality images.

### 2.1. Generating Adversarial Network (GAN)

Realistic image generation has become more and more important in computer graphics and multimedia studies. Creating realistic images requires fine-grained detail and high visual fidelity, which demands considerable time and computational resources. Nowadays, deep learning methods are widely used to produce high-quality photographs, especially generative models such as generative adversarial networks, or GANs [11]. Although GAN effectively addresses the challenge of obtaining picture data, it has unreliable training and produces images of low quality. An improved deep convolutional GAN with residual structures [12] aims to enhance training stability and image quality. To obtain the deep characteristics of actual picture samples, the network is deepened and image labeling information is incorporated using the residual structure. The discriminator model incorporates spectral limitations to achieve efficient picture data generation and enhance the network’s training stability. Experiments show that the recommended method outperforms the generated images in terms of their visual impact and objective evaluation. The LAPGAN model [13] is built by combining the GAN framework with a Laplacian pyramid, forming a new multiscale generation architecture. Over time, the model is able to transform low-resolution images into high-resolution ones. By taking use of the multiscale structure of real images, it creates several generative models, each of which may capture the image structure at a certain scale. At each size of the experiment, a convolutional network generative model is trained using the GAN technique. The sampling process starts from low-frequency residual images and gradually refines to the final image. Applications for CGAN [14] include the creation of synthetic data. For classification jobs, this produces data that are comparable to the original dataset while accounting for the category labels and making sure that there is as little correlation as possible between the created and original data. High-resolution, realistic images are produced using StackGAN++ [15], a novel class of stacked generative adversarial networks. The method consists of two versions: StackGAN-v1 and StackGAN-v2. For creating images from textual descriptions, StackGAN-v1 is a two-stage generative adversarial network that first produces a low-resolution image before producing a high-resolution picture. StackGAN-v2 is a more advanced multi-stage generative adversarial network that adopts a tree-like structure. It supports both conditional and unconditional image generation and produces images at multiple scales. By jointly approximating several correlated distributions, it improves training stability and overall image quality. According to experimental data, StackGAN++ performs noticeably better than other cutting-edge techniques at producing realistic images. Through progressive growth and multiscale statistical similarity assessment, a novel GAN training technique called PGGAN [16] dramatically increases the quality of the generated images. Its efficacy has been confirmed on several datasets. Additionally, CELEBA-HQ, an upgraded version of the CELEBA dataset, was created to enable experimentation at dimensions of up to 1024 × 1024 pixels. Even while their experimental results are of good photographic quality, they still lack true photo-realism, particularly when it comes to dataset-dependent restrictions and semantic understanding. StyleGAN [17], which is based on PGGAN, modifies the “style” at each convolutional layer to directly modify the intensity of image characteristics at various scales. Simultaneously, single-channel images with uncorrelated Gaussian noise are introduced as deliberately noisy inputs, giving the generator a way to produce random details directly. This suggests two novel automated techniques that can be applied to any generator architecture and are useful for measuring the quality of interpolation and decoupling. By lowering the variance of the generator inputs by orthogonal regularization of the generator, BigGAN [18] manages the trade-off between sample fidelity and diversity, improving the high fidelity of natural picture synthesis and making it appropriate for truncation approaches. The model was further evaluated on the JFT-300M dataset after being trained on ImageNet at resolutions of 128 × 128, 256 × 256, and 512 × 512. With a FID of 7.4 and an IS of 166.5 at 128 × 128 resolution, BigGAN outperforms the previous state-of-the-art models.

### 2.2. Diffusion Model

Compared to GAN, the diffusion model, a new picture generation mode, has a more precise log-likelihood computation and a more flexible model design. Some studies have improved the diffusion model. Nichol and Dhariwal [19] show that competitive log-likelihood performance can be achieved by making simple modifications to DDPMs while maintaining sample quality. They propose a hybrid learning objective that links a VLB and a simplification objective that achieves sampling with fewer forward pass steps. By learning the variance of the inverse process, their method significantly improves the sampling speed while maintaining sample quality, making the model more suitable for real-world deployment. Building on this line of improvement, Song et al. [20] introduced denoising diffusion implicit models (DDIMs), which preserve the training procedure of DDPMs but enable deterministic and accelerated sampling. With fast sampling, DDIMs provide both semantically rich latent interpolations and highly accurate image reconstructions. In their investigation of the use of diffusion modeling in text-conditional image synthesis, Alex Nichol et al. [21] contrasted two approaches: classifier-free guidance and CLIP guiding. They discovered that classifier-free bootstrapping frequently yielded realistic samples and was favored by human evaluators in terms of photo-realism and caption similarity. Furthermore, their model demonstrated the ability to perform effective text-driven image restoration and editing. A pre-trained language-image model (CLIP) [22] is combined with a DDPM that uses spatially mixed noise in the text-guided latent space [23]. This method enables local editing of natural images based on text descriptions and region of interest (ROI) masks, allowing edited regions to blend smoothly with the original image. Based on Ratcliff’s diffusion model, a diffusion model, Fast-dm, for reaction time (RT) analysis in binary decision-making tasks has been proposed [24]. The model uses a partial differential equation approach to quickly compute the predicted reaction time distribution. RT and error rate are then used simultaneously to avoid misleading speed-accuracy tradeoffs. The EZ-diffusion model, on the other hand, simplifies the parameter estimation process of the traditional diffusion model and is suitable for situations where the amount of data is small and the error rate is low [25]. It utilizes three observable variables—the mean RT, variance, and accuracy of the correct response—to calculate three key potential parameters. Fitting-free computation is achieved by displaying the equations instead of the iterative fitting process. The Q-diffusion method [26] achieves inference acceleration and memory compression by post-training quantization (PTQ) of a noisy estimation network in a diffusion model. The method identifies two major challenges in the quantization of diffusion models: large variations in activation distributions over multiple time steps and quantization errors accumulating over multiple inference steps. Two key techniques are then proposed to address these two problems: time-step-aware calibration data sampling and hop-connected channel separation quantization. The method can compress models such as stable diffusion [27] to 4-bit while maintaining high image quality. The aforementioned research enhances deep learning’s role in image generation by introducing efficient models and algorithms. This establishes a strong basis for further study and real-world applications.

### 2.3. Application of Artificial Intelligence in Traditional Culture Preservation and Design

Eyadah et al. [28] used analytical description methods as well as theoretical approaches, thus comprehensively and systematically classifying the application areas and development directions of artificial intelligence in cultural heritage protection. They aimed to develop a vision for the future that uses AI to preserve cultural assets. By employing artificial intelligence and machine learning for virtual restoration, Gaber et al. [29] contribute to the preservation of cultural assets and artifacts. They emphasize the potential and usefulness of artificial intelligence (AI) in preserving cultural heritage by discussing the significance of AI in this field, its current state of application, technological benefits, difficulties, and future development possibilities. At the same time, they combine a literature review and case study. The application of AI and ML in cultural heritage conservation, especially in virtual restoration, is studied. Meanwhile, combined with practical cases and content analysis, Zou et al. [30] comprehensively demonstrated the diverse applications of AI in cultural heritage protection, providing specific application scenarios and practical results. A qualitative research approach is used, which incorporates content analysis and case research. Examples are sourced from YouTube, the largest video website in the world, and well-known news websites (such as the BBC, CNN, The New York Times, and The Guardian). The interpretation of the gathered case texts serves as the foundation for the content analysis. The use of traditional culture in intelligent ad design systems in the Internet age was, however, investigated by Sun et al. [31]. An advertisement system model is presented, along with feature association algorithms and allocation algorithms for travel, quantity, and market rivalry in advertisement design. The background and importance of the combination of traditional culture and intelligent advertisement design systems are also discussed. Winiarti et al. [32] achieved cultural preservation using deep learning techniques by developing an application to identify traditional building types in Java, Indonesia. Their research included steps such as data collection, image analysis, application development, and evaluation; data collection was acquired through field photography and drones, and a total of 1330 images were collected. Test results showed that the system recognized architectural objects with 99.5% accuracy, and in education, the application was able to increase students’ knowledge of architectural history by 97%. Their AI-based technology provides an efficient tool for recognizing and learning traditional architecture. The study by Wu et al. [33] also looked at how traditional Chinese culture is being innovatively developed in artificial intelligence-based advertisement design. The study used artificial intelligence, artificial neural networks, extreme learning, and other methods to analyze the investment and development of traditional culture in advertising language. Contemporary ads perpetuate traditional Chinese culture by integrating its art forms, symbolism, aesthetics, and ideals through aesthetic analysis. Using the traditional ornamental patterns of ethnic minorities as the research object, Hu et al. [34] investigated the design technique under the trend of AIGC (Artificial Intelligence and Generative Design) in the context of globalization. In order to summarize the traits and traditional design principles of ethnic minorities, the traditional ornamental patterns of these groups are first arranged and examined. They then investigate how to use image computing and artificial intelligence in conjunction with AIGC-related technology to support the design process. Hang et al.’s study [35], on the other hand, explored the application of AI technology in packaging design in combination with traditional Chinese cultural and artistic elements. They analyzed how AI technology can support traditional Chinese cultural aesthetic packaging to meet modern consumer needs. Through a literature review, case studies, and conceptual framework, key challenges in commercialization are identified, and opportunities for sustainability and cross-cultural innovation are highlighted. In the context of wireless communication and artificial intelligence decision-making, Wu et al. [36] looked at ways to more effectively integrate traditional Chinese components into modern furniture design, mostly using surveys and sporadic telephone interviews. To understand public attitudes toward incorporating traditional elements into furniture, a questionnaire survey and random telephone interviews were conducted. The study also examined how these elements can be innovated through wireless communication and AI decision-making to better align with modern aesthetic concepts. AI decision-making and wireless connectivity provide new technical support and creative ideas for traditional furniture design. However, the research mainly focuses on the Northeast region, which may limit the representativeness of the sample. In addition, it relies primarily on questionnaires, lacking deeper analysis of user behavior and market feedback.

It can be seen that artificial intelligence technology can provide an innovative path for the protection and design of traditional culture. Through data collection and analysis, neural network generation, and other methods, artificial intelligence can significantly improve the design efficiency and creativity level of patterns. Its efficient data processing ability and analysis ability can enable us to better digitize the image and text information in traditional culture, and at the same time, can help us better understand traditional culture. It is known that the cross-application of traditional culture and artificial intelligence is extremely necessary. In addition, it can be seen that the diffusion model has been widely used in research on image generation. Moreover, in research on text-generated images, compared with the traditional GAN model, it has superior performance and can efficiently generate high-quality images. However, the computational complexity of the diffusion model is high, which consumes considerable training time when it is used to deal with large-scale datasets, and it is challenging to use it on the large dataset of Dunhuang patterns in this paper. Moreover, the diffusion model has a high degree of modeling requirement for the training data, which will affect the results of the generated images when the output data are unbalanced after training. There are many challenges in the application of Dunhuang traditional patterns in modern design, such as the possible loss of symbolic semantics or the distortion of visual features when using artificial intelligence technology to form Dunhuang patterns. Therefore, we propose a new DANet to solve the difficulties in generating Dunhuang patterns.

Although most of the current research focuses on the digital reconstruction and generation of local cultural patterns, with the increase in the generalization of AI-generated models, how to adapt the models to traditional visual elements in different linguistic and cultural contexts has become an important direction for future research. Therefore, based on our exploration of Chinese Dunhuang patterns, we also realize the potential adaptability and expansion space of this method in multilingual contexts and multicultural visual systems. For example, the sketch + semantic description fusion generation mechanism adopted in this paper is not limited to the semantics of Chinese or Chinese characters. Its structural cueing and image semantic guidance mechanisms can be generalized to other cultural systems, such as Indian Buddhist imagery, Central Asian mural painting styles, and even the visual expression systems of non-written cultural heritage. This multimodal generation approach offers the possibility of establishing a more universal cultural generation framework and opens up new paths for the study of traditional pattern generation in non-Chianese contexts.

## 3. Methodology

### 3.1. Overall Structure of DANet

**Figure 1:**
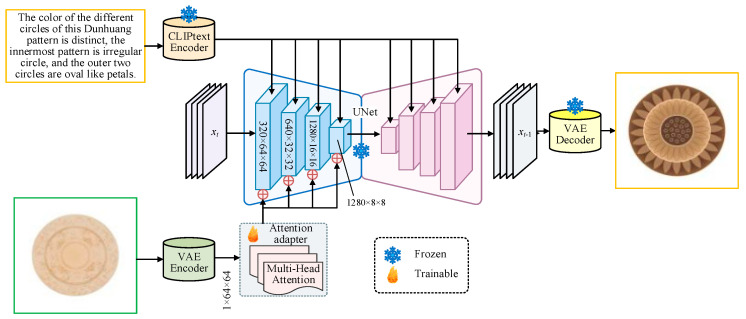
Overall structure of the DANet.

**Figure 2:**
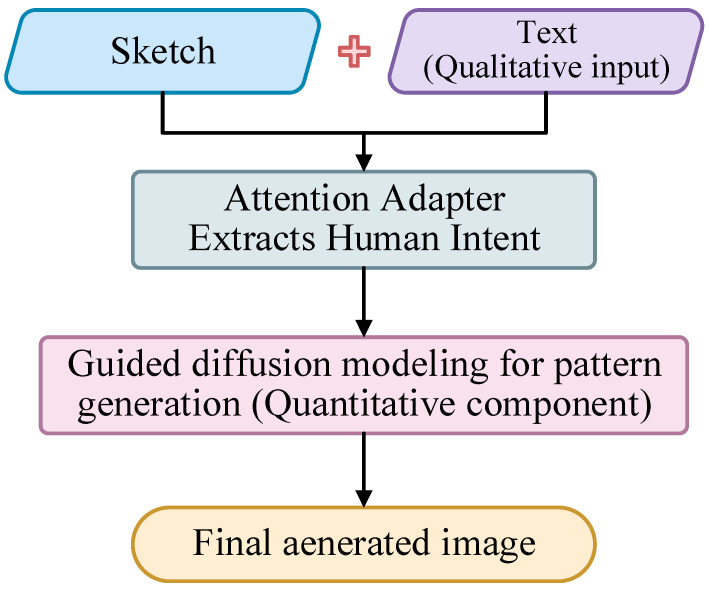
Qualitative-quantitative fusion flowchart.

The overall structure of the DANet is shown in Figure 1. Based on the diffusion macromodel SD1V5, this paper further proposes an attention adapter, which can realize multimodal generation of Dunhuang patterns by fusing text information, sketch structure, and other conditional information. Firstly, we input text information and sketch data (Table 1) and process the text description using CLIP text encoder, while the sketch data are processed by VAE encoder. The processed sketch data are trained using the attention adapter, in which the multihead attention module (MHAM) is used to enhance the cue information of image modality, the MSAM module features images at different resolutions for more comprehensive image understanding, and the ACM module adaptively adjusts and optimizes the model mechanism. After further processing, the image and text information are feature extracted and processed through the UNet structure. Finally, the VAE decoder is utilized to output our desired Dunhuang patterns (Figure 2). In this process, there are freezing parameters in the CLIP text encoder and VAE decoder, which indicate the parts that remain unchanged during training. There are trainable parameters in the attention adapter that represent the update of sketch modality.

To address the limited semantic understanding of Dunhuang patterns caused by freezing the parameters of the pre-trained diffusion model, we enhance the attention adapter’s ability to capture domain-specific features in two key ways. Specifically, we introduce a multihead attention module (MHAM) and a multiscale attention module (MSAM) to effectively extract structural and fine-grained information from both sketches and textual cues. MHAM improves semantic focus by attending to multiple subspaces in parallel, enabling the extraction of pattern-critical features. Meanwhile, MSAM ensures the perception of details across different spatial scales. Together, these modules strengthen the adapter’s capacity to perceive and represent the distinctive characteristics of Dunhuang patterns. Furthermore, we introduce a semantic classification supervision mechanism by incorporating a cross-entropy loss during the training of the attention adapter. Given the diverse themes and stylistic variations in the Dunhuang pattern dataset, this supervision encourages the model to perform fine-grained semantic discrimination in the feature space. As a result, the model becomes more capable of distinguishing and understanding the specific semantics associated with each pattern type. This enhances the adapter’s representational precision. Notably, since the underlying diffusion model has been pre-trained on large-scale image–text pairs, it already possesses strong visual-linguistic understanding, which our method effectively leverages. Based on this, the unique semantics of Dunhuang patterns can be effectively captured by training a lightweight adapter on the dataset proposed in this paper. This approach efficiently leverages the visual-linguistic knowledge and structural priors of the pre-trained model, enabling fast and economical adaptation to Dunhuang-specific features. In addition, the generalized visual-semantic knowledge acquired through the pre-training of the base model is retained and remains robust against interference from limited domain-specific data. This ensures that the generated patterns maintain both broad generalization and distinct Dunhuang-specific characteristics. As a result, it strikes a practical balance between computational cost, training efficiency, and generation quality.

From a design perspective, the DANet model accurately generates culturally accurate traditional patterns by fusing sketches and textual inputs. This approach not only respects the designer’s creative input (e.g., initial sketches or conceptual descriptions), but also provides a new design tool in the creative design process: designers can efficiently visualize and explore cultural symbols. Especially in traditional pattern design, the accurate expression of the cultural semantics and formal characteristics behind the patterns is crucial. By precisely regulating the attention mechanisms, DANet enables designers to better control cultural fidelity and visual expression in the creative process. This effectively enhances the integration of design creativity and cultural meaning.

### 3.2. Attention Adapter

**Figure 3:**
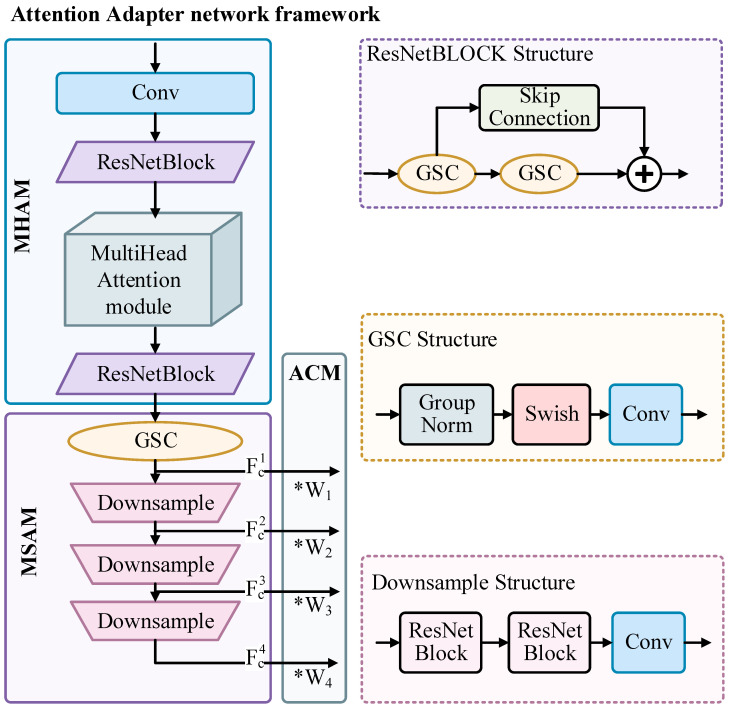
Structure of Attention Adapter.

We first utilize the multihead attention module (MHAM) to process the input sketch. After many attempts, this method chooses the 8-head attention mechanism to divide its channels into 8 attention heads. The 8-head attention mechanism enables the model to attend to multiple feature subspaces simultaneously. Moreover, it allows for an even distribution of channel dimensions, preventing over- or under-partitioning that may result in unnecessary computational overhead. Given the query features, the output of multihead attention can be calculated by Equations (1) and (2),

(1) $$X^{\prime }=Attention\left(S,\;O,V\right)=softmax\left(\frac{SO^{T}}{\sqrt{d}}\right)\cdot V$$

(2) $$\begin{array}{c} MultiHead\left(S,O,V\right)=Concat\left({head}_{1},\ldots ,{head}_{h}\right)W^{P}\\ where\;{head}_{i}=Attention(SW_{i}^{S},OW_{i}^{O},VW_{i}^{V}) \end{array}$$

where $S=XW_{s}$, $O=XW_{o}$, $V=XW_{v}$, are the query, key, and value matrices of the attention operation, respectively, $W_{s}$, $W_{o}$, $W_{v}$ are the weight matrices of the trainable linear projection layer, and $W^{P}$ is the learnable linear transformation matrix. Based on this design, each head may focus on a different part of the input and can represent more complex functions than weighted averages. (See Figure 3).

**Figure 4:**
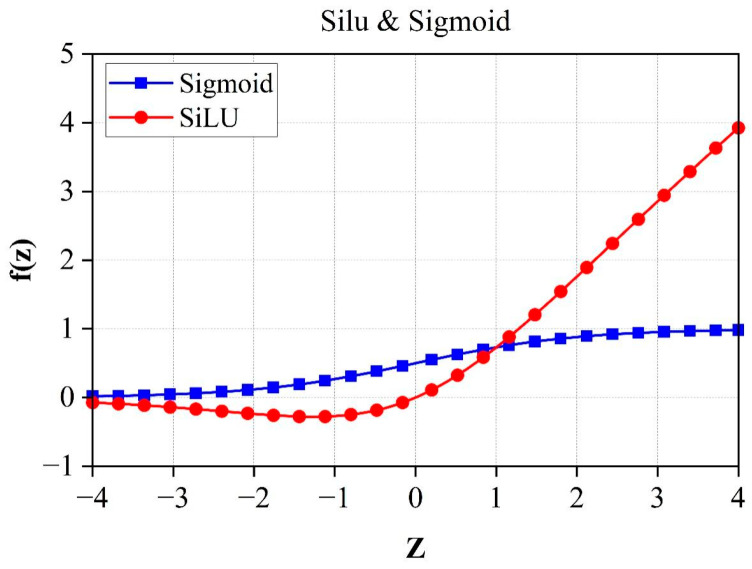
Swish vs. Sigmoid.

Following the multihead attention module (MSAM), the output features are processed by a GSC layer and subsequently passed through three residual downsampling blocks; this sequence facilitates the extraction of multiscale features. The GSC layers are group normalization (GN), SiLU activation, and convolutional fusion, which extend the number of feature channels with the same size to 320. The GN layer splits features into eight groups and applies mean-variance normalization within each group. This design mitigates the influence of varying batch sizes and ensures stable training even under small-batch conditions. It then goes through the Swish function in the form of Equation (3). As shown in Figure 4, this activation function provides a smoother gradient flow compared to the traditional RLU activation function, which helps to improve the training process and performance of the deep learning model. Finally, a specific convolutional layer is used to maintain the output size after convolution. This allows the network to capture more detailed features and enhances the model’s generalization and performance.

(3) $$f\left(z\right)=z\times sigmoid(z)$$

The features are then passed through a downsampling block to extract additional multiscale information. By progressively reducing the spatial dimensions, the model captures features at different scales, enhancing its perception and improving generalization. Each of these downsampling blocks is designed with a similar structure, consisting of two residual network layers and a downsampling convolutional layer, all with a downsampling rate of 2. After a layer of downsampling, the feature size is reduced by a factor of 2, and there are differences only in the input and output channels, and the output features are in the order of 640, 1280, and 1280. The process of compressing the feature $F_{C}^{1}$ of 64 × 64 size into a feature $F_{C}^{4}$ of 8 × 8 size again forms a multiscale feature, $F_{C}=\{F_{C}^{1},F_{C}^{2},F_{C}^{3},F_{C}^{4}\}$. $F_{C}$ has the same size as the intermediate feature $F_{enc}$ of the diffusion model and is finally added one by one at the same scale.

In the adaptive conditioning mechanism (ACM), there are four levels of intermediate features in the downsampling process of the UNet encoder, and the features of different layers have different impacts on the generation results. Therefore, this study introduces an adaptive adjustment mechanism for the conditional guidance weights of each feature layer. During training, the network automatically adjusts these weights via backpropagation, enabling the attention adapter to flexibly fuse features. This ensures effective structural control using various inputs such as sketches and text. In summary, multiscale feature extraction and conditional control can be defined as Equations (4) and (5),

(4) $$F_{C}=Fma(∁)$$

(5) $${{\bar{F}}^{i}}_{enc}=F_{enc}^{i}+W_{i}\times F_{C}^{i},\;i\in \{1,\;2,\;3,\;4\}$$

where *C* represents the features extracted by the VAE, Fma represents the adapter network, $F_{C}^{i}$ represents the multiple intermediate features extracted, $W_{i}$ represents the corresponding different weights, and $F_{enc}^{i}$ is each intermediate layer feature.

### 3.3. Cross-Entropy Loss

During the optimization process, the SD1V5 parameters are frozen and only the attention adapter is trained, using the same optimization objective as SD1V5. Each training sample is a ternary consisting of the original image Z, the condition map C, and the prompt text *y*. Given an image Z, it is first embedded into the potential space X by the encoder of the VAE. Then, a time step t long, X, is randomly sampled from [0,T] and the corresponding noise is added to obtain $X_{t}$. The noise prediction network is fixed by Equation (6) to optimize the ability of the modal information extracted by the attention adapter through the prediction of the noise residuals, which is denoted as φ, the domain-specific encoder; $F_{C}$ is the modal cue information, $h_{\theta }$ is the noise prediction network, and h is the actual noise. If this function is weighted, which is achieved by introducing a weighting factor , the weighted loss function can be expressed as Equation (7), where θ is a scalar greater than 0 used to adjust the weights of the loss function.

(6) $$L_{ma}={Ε}_{X_{0},t,F_{C},h\sim N(0,1)}[{∥h-h_{\theta }\left(X_{t},t,\varphi \left(y\right),F_{C}\right)∥}_{2}^{2}]$$

(7) $$L_{ma}^{\theta }={\theta \cdot Ε}_{X_{0},t,F_{C},h\sim N(0,1)}[{∥h-h_{\theta }\left(X_{t},t,\varphi \left(y\right),F_{C}\right)∥}_{2}^{2}]$$

In diffusion modeling, temporal embedding is an important condition for sampling. The main content of the generated results is determined in the early sampling stage, the features are inserted into the first feature fusion stage, and the t-distribution of the cubic function $t=\left(1-{\left(\frac{t}{T}\right)}^{3}\right)\times T,t\in (0,T)$ is used to increase the probability of the pre-sampling drop. Because the noise intensity of randomly sampled images may be uncontrolled, in this paper, the noise is regularized by dividing it by the variance to prevent the noise intensity from exceeding the bounds, the predicted image is then obtained by directly dividing the noise, and the difference between the original image and the predicted image is then calculated.

## 4. Experiments

### 4.1. Dataset and Metrics

**Figure 5:**
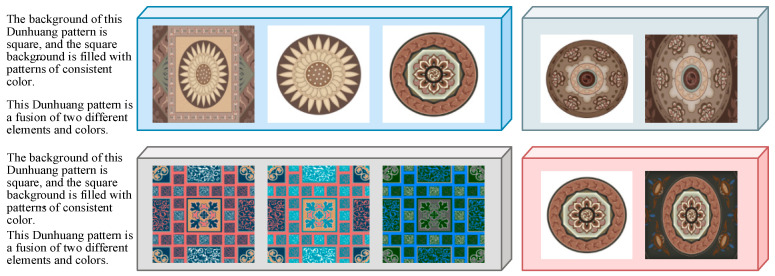
Schematic diagram of the DDHP dataset.

**Figure 6:**
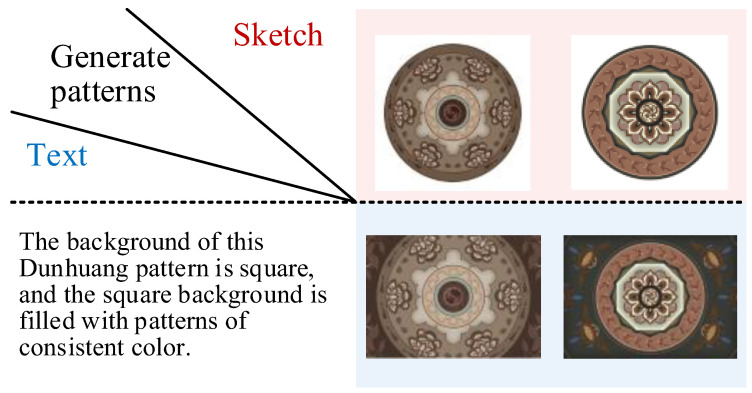
Multimodal information cross-generation effects generated by single sketch and text.

**Figure 7:**
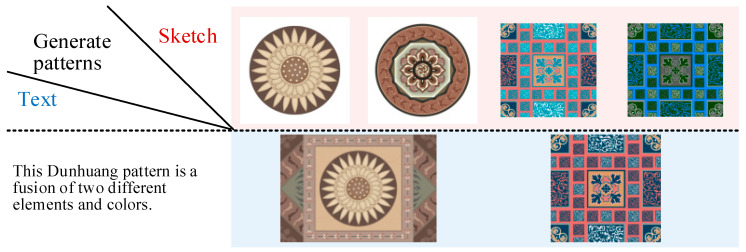
Multimodal information cross-generation effects generated by multi-sketch and text.

In this paper, we build our own Dunhuang patterns image dataset DDHP, which has 3000 pattern images divided into 2800 training sets and 200 test sets, as shown in Figure 5, and each image has semantic masks, sketches, descriptive texts, and images with different backgrounds. This method uses its pattern images, sketches, and texts, crops the images to 512 × 512 size, and randomly selects corresponding pattern images from 10 articles in this description to enhance the text diversity. Due to the specificity of the patterns, the experiments use the conditions of the sketch for multimodal pattern generation. Figure 6 and Figure 7 show the effect of the mixed cross-generation of textual data and modeling information, where the images on the diagonal line are the results of the matching textual modal pairs of guided generation, and the rest are the results of the confusing guided generation. The 3000 hand-drawn sketches represent the basic structural features of Dunhuang motifs, and each sketch (Table 2) is accompanied by a hand-written descriptive text covering the elements of the motifs, stylistic imagery, and cultural meanings, such as “Flying maidens encircling a lotus flower center,” “Lianzhu pattern combined with a flame totem structure,” and so on. These texts were compiled by design researchers with reference to the classification of Dunhuang motifs.

In this paper, the validation is carried out on the test set of DDHP, whereby 2000 patterned graphic pairs of the test set are selected for validation testing, and the original images, sketches, and descriptive texts are chosen, and for each sample, different models are randomly sampled once as the final results. The inter-image similarity evaluation index typically uses the perceptual similarity LPIPS column (learned perceptual image patch similarity), while graphic matching and image feature matching of the generated results are used as evaluation indexes, with the graphic matching degree CLIP score (ViT-L/14) and feature similarity CLIP-I used, respectively, to qualitatively evaluate the generation effect of the attention adapter model. The LPIPS is calculated as shown in Equation (8),

(8) dz,z0=∑l1HlWl∑h,w∥Wl⨀(yhw^ll−y0hw^l)∥22

where z generates the image, $z_{0}$ is the original image, and $\hat{y_{hw}}$ is the neural network that extracts the output of each layer for activation after normalization of the features. This paper uses the AlexNet network, and after w layers of weights re-multiplication, it calculates the L2 distance and then averages it. A lower score indicates that the quality of the generated image is closer to the original image.

Since the LPIPS score does not measure whether the generated image matches the text description and modal information cues, this paper adopts the CLIP score and CLIP-I as the evaluation metrics. The CLIP score uses the CLIP model to extract and measure the cosine similarity between the text features and the image features to evaluate the graphic matching, which is computed as shown in Equation (9),

(9) $$CLIPScore\left(I,C\right)=max\;(W\times \cos\left(G_{l},G_{c}\right),0)$$

where $G_{c}$ and $G_{l}$ are the features output by the CLIP encoder for text and image processing, W is a constant taking the value of 2.5, and a higher CLIP score represents a better consistency between the generated image and the text.

CLPI calculates the feature similarity between the generated image and the cued image to evaluate the similarity and is calculated as shown in Equation (10),

(10) $$CLIP-I\left(I_{1},I_{2}\right)=max\;(\cos\left(I_{1},I_{2}\right),0)$$

where $I_{1}$ and $I_{2}$ represent the features of the generated image and the cued legend, respectively, and a higher CLIP-I represents the more similar features of both.

### 4.2. Ablation Experiments

In this paper, two guided generation methods, sketch and text, are selected for multimodal Dunhuang pattern generation. Using 2000 images from the DDHP test set for the evaluation, for each image, different methods are randomly extrapolated only once as the final result. For the effect of the model in generating Chinese traditional pattern graphics, the analysis of the experimental results will be unfolded from the ablation experiments in the following.

To verify the effectiveness of each module, this paper adds each module of the attention adapter one by one on the basis of the SD1V5 model and constructs ablation experiments on the DDHP test set. For a fair comparison, the ablation experiments use the same training configuration and number of iteration steps, and the results are shown in Table 3.

Compared with the base pedestal model, the model gains about an 8% improvement in the CLIP-I score after adding the MHAM module, indicating that the multihead attention mechanism helps to improve the model’s ability to extract and fuse key features. In addition, the base model with the MSAM module gains about 7.3% improvement in the CLIP-I score, indicating that multiscale feature injection fusion is not only structurally aware of model generation but also contributes to graphical consistency. When the ACM module is introduced on this basis, both the CLIP score and CLIP-I score indicators are partially improved, indicating that the ACM module can optimize the feature adjustment weights to effectively improve the effect. When both the MHAM module and MSAM model are introduced to the base model, the scoring performance is improved, indicating that the two modules are complementary to each other to promote performance improvement. Finally, when using three modules, that is, the complete attention adapter model, the best performance is achieved in the CLIP score and CLIP-I scoring indexes, which indicates that the other two modules together with the ACM module can adaptively adjust the weights of different feature injection layers to obtain better results.

In addition, to evaluate the effectiveness of semantic supervision using cross-entropy loss, we compare it with a variant of the DANet trained using L1 loss. The results show that the DANet (L1 loss) achieves a CLIP score of 0.521 and a CLIP-I of 0.745. As a pixel-level supervision signal, L1 loss emphasizes low-level detail reconstruction rather than high-level semantic categorization. While this may improve the realism of generated images at the pixel level, it often leads to weaker semantic consistency in the generated patterns. The experimental results show that DANet supervised with cross-entropy loss achieves higher CLIP score and CLIP-I values compared to L1 loss. This indicates better performance in terms of semantic and categorical accuracy. Cross-entropy loss provides more effective supervision for the attention adapter, enhancing its ability to capture Dunhuang-specific semantics and significantly improving the consistency and accuracy of the generated patterns.

### 4.3. Comparative Experiments

For a fair comparison, all the compared models are fine-tuned on the DDHP training set. In the case of collaborative diffusion, the same training data are used as in this paper, but without additional fine-tuning. Under the same conditions, each method randomly generates 1 image per input, and the evaluation metrics are computed as the average of over 2000 generated images. We compare our proposed method with several representative mainstream approaches, including collaborative diffusion, the original SD1.5, ControlNet, as well as GAN- and CGAN-based methods that rely on the efficient fine-tuning of large models. In addition, to evaluate the semantic modeling and generation capability of our proposed adapter for Dunhuang images under a frozen large-scale diffusion model, we conducted a comparison with the diffusion model fine-tuned using LoRA on SD1.5. LoRA achieves lightweight fine-tuning by introducing low-rank matrices to a subset of parameters, which is similar in spirit to the training strategy used by DANet.

In the DDHP dataset, each input (textual description as well as the corresponding sketch) is accompanied by a real target pattern (i.e., Ground Truth). These images are used in both the training and evaluation of GAN-like models. For diffusion-based models, the method is used as much as possible in the evaluation phase. The performance comparison results of the DANet with current mainstream methods are shown in Table 4. Its score reaches the best in terms of LPIPS score performance, especially for the image of Dunhuang patterns generated by using sketch guidance, which is reduced from 0.532 to 0.498, with a performance improvement of about 6.4% compared to the GAN and about 31.7% compared to the CGAN. This fully indicates that the generated pattern guided by the DANet model is most similar to the original image. It performs better in guiding image generation when structural information is available. Moreover, it can more accurately guide large models to generate images that meet multimodal constraints.

In this paper, we use the CLIP model to compute the generated image and modal information feature consistency, and the performance of the CLIP score shows that the DANet performs the best and the GAN scores the lowest. This indicates that compared with the conventional diffusion model dedicated to images, the generalized diffusion macromodel trained from massive data has a strong generative ability and significantly outperforms the GAN model in the graphic matching score. Compared to the CGAN, the DANet model score grows from 0.512 to 0.533, with a performance improvement of about 4.1%. The improvement is about 33.9% compared to the GAN, and the DANet score also achieves some improvement compared to other mainstream methods, showing the best performance. This indicates that the DANet performs better in terms of graphical consistency between the prompt text and the generated image on pattern image generation, and it can better balance the textual semantic description and structural modal information to guide the generation of the corresponding pattern features. From the CLIP-I score measurements, the DANet achieves the highest score superior to other mainstream methods. Based on the sketch generation effect compared with the GAN, the CLIP-I score improves from 0.649 to 0.772, which is a performance improvement of about 18.9% and a performance improvement of about 14.9% compared with the CGAN. This reflects that the DANet-guided generated pattern image is not only similar in pixel space to the original pattern image providing structural modal information but also more similar in the depth features extracted by CLIP, and the DANet can more effectively utilize the depth features of structural modal information for guided image generation. In conclusion, the DANet model proposed in this paper can fully utilize the conditional information and achieve the best results in all the tested metrics.

In addition, from Table 4, it can be seen that in terms of image generation efficiency, all experiments were inferred on a single NVIDIA A100 GPU (40 GB). The GAN-based method has a very high inference speed because the generation process relies on only one forward propagation without multi-step iterative sampling. Its FPS ranges from 1.8 to 12.5, and the inference speed gradually becomes slower as the network structure becomes more complex. Traditional GANs have an FPS of up to 12.5, while BigGANs and CGANs with more parameters have an FPS of 2.2 and 1.8, respectively. Compared to GANs, the diffusion model-based approach has a slower inference speed. It needs to generate images through a sampling process of gradual denoising, so the inference speed is limited. In order to generate higher-quality Dunhuang images, the DDIM sampler is used in the experiments, and the number of sampling steps is set to 50. It can be seen that the diffusion model has an FPS of 0.52, and the LoRA-Baseline based on diffusion fine-tuning has an FPS of 0.50. The DANet’s inference speed decreases compared to the previous two due to the introduction of the additional attention adapter structure, which is 0.42. LoRA just merges additional small matrices and thus hardly increases the inference cost of the diffusion model. It improves the CLIP Score and CLIP-I of SD while ensuring that the inference speed is not reduced. The attention adapter is an additional module that targets the Dunhuang pattern generation task by utilizing multihead attention and multiscale features to extract key information introduced from the outside, instead of only low-rank updates to the original model parameters. Cross-entropy loss is also specifically introduced to categorize the semantics. As a result, its inference speed is lower than LoRA-diffusion. However, it can be seen that for Dunhuang patterns, the DANet is more expressive in terms of semantic guidance and stylistic consistency. This proves that the performance of the attention adapter is improved before keeping the computational overhead low.

Table 5 shows the visualization results of the DANet, PGGAN, and LoRA-Baseline under the joint guidance of sketches and text. Among them, the first column is the sketch drawn manually, and the second column is the text description used for semantic guidance. It can be seen that the DANet better preserves the structural details in the sketches, such as the petal shape of the lotus flower and the hand connection relationship of the flying dancer. At the same time, it is able to creatively deal with the color layer changes of the lotus flower to make it look more vivid. The generation effect of LoRA-Baseline can be seen from its poor understanding of semantics, failing to capture the context of Dunhuang to design the sketch. In contrast, the PGGAN creates a more cartoonish image of the dancing girl drawing style that originally symbolizes the characteristics of Dunhuang. This suggests that the DANet performs better in stylistic consistency and spatial semantic alignment. It is able to more accurately realize the Dunhuang-style compositions described in the text. The attention adapter proposed in the DANet is superior in the integration and expression of Dunhuang pattern styles.

## 5. Conclusions

In this paper, DANet and cross-entropy loss are proposed to realize the multimodal cueing pattern picture capability of the diffusion macromodel of Vincennes images. The core design of the attention adapter proposed in this paper is based on a multihead attention strategy and a multiscale attention module. These modules extract key visual modal cues and multiscale features for multimodal attention, while the diffusion-based large model provides structural guidance to enable the accurate generation of Dunhuang patterns. Furthermore, in the DANet in this paper, the cross-entropy loss function is introduced. The Softmax function is first used to convert the output of the model into a probability distribution, and then the cross-entropy loss function is used to measure the difference between this predicted probability distribution and the true probability distribution. This combination not only makes it easy to calculate the gradient but also provides good numerical stability during the optimization process. The results of the ablation and comparison experiments demonstrate that the proposed attention adapter outperforms mainstream models in both the semantic guidance and structural consistency of image generation. In addition, it consumes fewer training resources than the other comparison models. The attention adapter proposed in this study demonstrates strong generality and generalization ability. It can guide exemplar generation by combining multiple attention modules. It is not only compatible with custom models in the same architecture but also integrates seamlessly with existing style-controlled generation tools. This feature makes the attention adapter not only suitable for the image generation of Dunhuang patterns but also can be widely used to guide the generation of general-purpose objects, which broadens its scope of application. In the aspect of design theory, this study for the first time integrates qualitative design methods with quantitative deep learning techniques, proposing a new way to empower design creativity with technology.

Despite the above contributions of the model proposed in this paper, it is currently mainly targeted at Dunhuang motifs and has not yet been validated on other traditional arts. In addition, the use of English text cues may limit the direct application of the model in Chinese scenarios, requiring users to have a certain level of English proficiency. The improvement based on the diffusion model makes the inference of the DANet slower; although within acceptable limits, it still needs to be improved in practical applications.

From a design methodological perspective, this study is the first to integrate qualitative design approaches with quantitative deep learning techniques, presenting a novel framework for enhancing creative design through technological means. At the same time, it also provides a new vision and practice example for future research on the cross-fertilization of design theory and intelligent technology. However, at present, this method mainly uses English text prompts, and the pattern images generated are mainly Dunhuang patterns. It is still necessary to adjust the model for the demands of the Chinese environment and generating images of Dunhuang patterns, and there may be some limitations in applying it to related fields in China. In the future, our goal is to explore how to apply this technology more effectively in domestic public security-related fields, try to integrate more dimensions of modal information fusion to guide image generation, change the language of textual modal information, replace English with Chinese descriptions, optimize the base generation model, use the base model of the Dunhuang patterns for generation, and fine-tune the base model to achieve more practical applications.

## Figures and Tables

**Table 1:** Input sample diagram.

Sketch	Text	Output
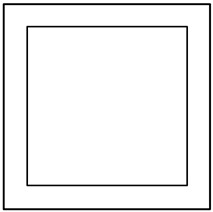	Flying fairies with a blend of lotus flowers and auspicious clouds.	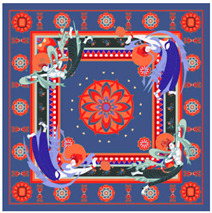

**Table 2:** Sample sketches and text presentation.

Sketch	Text	Statement of Design Intent
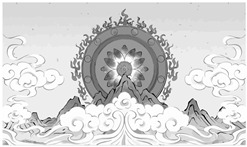	A lotus in full bloom, blended with auspicious clouds and flames, symbolizing euphoria and prosperity	The image tries to capture the “aesthetics of the bloom”, reflecting the dynamics of the blossom.
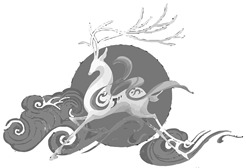	A white deer with long antlers all the time, surrounded by flowing clouds, symbolizing good luck and beautyThis image shows a mythical deer with an elegant stance and long antlers.	Trying to capture the “aesthetics of running”, reflecting the momentum of jumping.
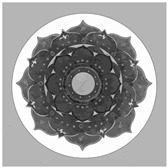	A lifelike lotus flower whose petals are partially fused with auspicious clouds, symbolizing good luck and good fortune.	The image attempts to capture the “dynamic aesthetics of the bloom,” reflecting the dynamism of the lotus bloom.

**Table 3:** Results of ablation experiments on the DDHP dataset.

Combination	MHAM	MSAM	ACM	Cross-Entropy Loss	L1 Loss	CLIP Score ↑	CLIP-I↑
None	×	×	×	√	×	0.473	0.649
MHAM	√	×	×	√		0.485	0.701
MSAM	×	√	×	√		0.499	0.697
MSAM-ACM	×	√	√	√		0.505	0.699
MHAM-MSAM	√	√	×	√		0.514	0.758
DANet (L1 Loss)	√	√	√		√	0.521	0.745
**DANet**	√	√	√	√		**0.532**	**0.774**

**Table 4:** Comparative experimental results on the DDHP dataset.

Methods	Number of Training Parameters	LPIPS-Alex ↓	CLIP Score ↑	CLIP-I ↑	FPS
GAN	23M	0.532	0.398	0.649	12.5
LAPGAN	28M	0.568	0.431	0.656	8.7
StackGAN++	44M	0.612	0.463	0.701	4.9
PGGAN	37M	0.633	0.482	0.698	5.6
StyleGAN	62M	0.691	0.501	0.675	3.4
BigGAN	73M	0.704	0.483	0.712	2.2
CGAN	91M	0.729	0.512	0.734	1.8
Diffusion	845M	0.834	0.479	0.672	0.52
LoRA-Baseline	12M	0.513	0.512	0.751	0.50
**DANet (Ours)**	**94M**	**0.498**	**0.532**	**0.774**	**0.42**

**Table 5:** Visualization results for different models.

Sketch	Text	PGGAN	LoRA-Baseline	DANet
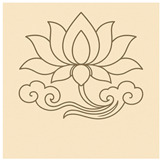	A Dunhuang-style lotus pattern with cloud motifs, viewed from the front.	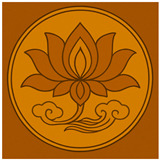	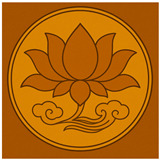	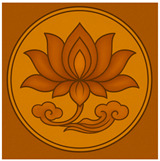
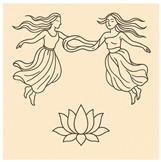	Two dancing fairies in Dunhuang mural style holding hands above a lotus flower.	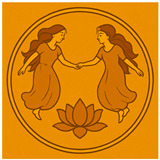	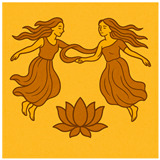	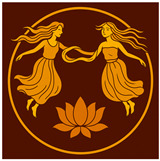

## Data Availability

All data generated or analyzed during this study are included in this article. The raw data are available from the corresponding author upon reasonable request.
